# Caesarean section among referred and self-referred birthing women: a cohort study from a tertiary hospital, northeastern Tanzania

**DOI:** 10.1186/1471-2393-11-55

**Published:** 2011-07-28

**Authors:** Ingvil K Sørbye, Siri Vangen, Olola Oneko, Johanne Sundby, Per Bergsjø

**Affiliations:** 1 National Resource Centre for Women's Health, Department of Obstetrics and Gynaecology, Oslo University Hospital HF, Rikshospitalet, Oslo, Norway; 2 Norwegian Institute of Public Health, Oslo, Norway; 3 Kilimanjaro Christian Medical Centre, Department of Obstetrics and Gynaecology, Moshi, Tanzania; 4 Institute of Health and Society, Department of International Community Health, University of Oslo, Norway

## Abstract

**Background:**

The inequity in emergency obstetric care access in Tanzania is unsatisfactory. Despite an existing national obstetric referral system, many birthing women bypass referring facilities and go directly to higher-level care centres. We wanted to compare Caesarean section (CS) rates among women formally referred to a tertiary care centre versus self-referred women, and to assess the effect of referral status on adverse outcomes after CS.

**Methods:**

We used data from 21,011 deliveries, drawn from the birth registry of a tertiary hospital in northeastern Tanzania, during 2000-07. Referral status was categorized as self-referred if the woman had bypassed or not accessed referral, or formally-referred if referred by a health worker. Because CS indications were insufficiently registered, we applied the Ten-Group Classification System to determine the CS rate by obstetric group and referral status. Associations between referral status and adverse outcomes after CS delivery were analysed using multiple regression models. Outcome measures were CS, maternal death, obstetric haemorrhage ≥ 750 mL, postpartum stay > 9 days, neonatal death, Apgar score < 7 at 5 min and neonatal ward transfer.

**Results:**

Referral status contributed substantially to the CS rate, which was 55.0% in formally-referred and 26.9% in self-referred birthing women. In both groups, term nulliparous singleton cephalic pregnancies and women with previous scar(s) constituted two thirds of CS deliveries. Low Apgar score (adjusted OR 1.42, 95% CI 1.09-1.86) and neonatal ward transfer (adjusted OR 1.18, 95% CI 1.04-1.35) were significantly associated with formal referral. Early neonatal death rates after CS were 1.6% in babies of formally-referred versus 1.2% in babies of self-referred birthing women, a non-significant difference after adjusting for confounding factors (adjusted OR 1.37, 95% CI 0.87-2.16). Absolute neonatal death rates were > 2% after CS in breech, multiple gestation and preterm deliveries in both referral groups.

**Conclusions:**

Women referred for delivery had higher CS rates and poorer neonatal outcomes, suggesting that the formal referral system successfully identifies high-risk birth, although low volume suggests underutilization. High absolute rates of post-CS adverse outcomes among breech, multiple gestation and preterm deliveries suggest the need to target self-referred birthing women for earlier professional intrapartum care.

## Background

Progress towards Millennium Development Goals (MDGs) 4 and 5, to reduce child and maternal mortality and morbidity, is unsatisfactory in most sub-Saharan countries [1,2]. It is widely acknowledged that improved and equal access to emergency obstetric care is crucial in addressing survival [3-5]. As is the case for several sub-Saharan countries, Tanzania has had a constant low national average Caesarean section (CS) rate (3%) since 1999 [6]. This is below the minimum recommended level, reflecting considerable unmet needs for emergency obstetric care [7-9]. Where a large proportion of births take place outside facilities, an effective referral system is necessary, but not sufficient, to achieve equitable access to emergency obstetric care. Normal delivery services are available in 74% of health facilities in Tanzania, but only 40% of facilities provide emergency transport to a referral site where comprehensive emergency obstetric care is offered [10]. Although a national referral system with standard criteria for referral of obstetric complications in women in need of hospital delivery is implemented in Tanzania, bypass of referring facilities ('self-referral') is a familiar phenomenon [11,12].

In the Kilimanjaro Region in the Northern Zone of Tanzania, the CS rate is 7.2% [6]. Although more than double the national average, the rate does not disclose whether or not the interventions target those who need them and is no guarantee for equity in access to care [13]. Our study was initiated by the findings that the zonal tertiary hospital, Kilimanjaro Christian Medical Centre (KCMC), had a high CS rate of 33% during the period 2000-07, together with a high proportion of self-referred birthing women. We wanted to compare CS rates among women formally referred for hospital delivery versus self-referred women. A secondary objective was to assess risk of adverse maternal and neonatal outcomes after CS according to referral status. As indications for CS were insufficiently registered, we applied the Ten-Group Classification System [14] to identify risk groups for targeted intervention.

## Methods

### Study setting

We used data from the medical birth registry at the zonal referral hospital KCMC in northeastern Tanzania to perform a cohort study of 21,011 births and 21,614 newborns from the period January 1^st ^2000 to August 31^st ^2007. Births with birth weight ≥ 500 g or gestational age ≥ 22 weeks were included. The birth registry, which has been described in detail elsewhere [15], systematically and prospectively collects information on sociodemographic and basic obstetric indicators, as well as information on delivery modes and pregnancy outcomes. Trained midwives conduct interviews and collect case record information in the days after birth, with a response rate of > 98%. The facility runs as a private/public partnership. The obstetric department receives patients from the local uptake area (Moshi town) in addition to referrals from a larger geographical area. CS is almost exclusively performed at hospitals in Tanzania [10], and most CS deliveries for women living in urban Moshi (Moshi District Council) are carried out at the facility. In Kilimanjaro Region, 70% of births take place at a health facility [6], and in Moshi, 92% deliver at a facility [16]. The site of the present study (a tertiary birth centre) is thus not a population-representative sample, as many women deliver at lower level facilities in the area or at home. There is potential selection towards financially better off women due to the cost-sharing policy gradually introduced for maternity services at KCMC from 2005 onwards. For a normal delivery, out-of-pocket costs are in the range of 5,000-15,000 TZS (5- 15 USD), while a CS has added minimum costs of 25,000-30,000 TZS (25-30 USD) [17]. In comparison, 88.5% of the population in Tanzania lived on less than USD 1.25 a day in 2000 [18]. The national health policy provides exemptions for the poor, but these are incompletely implemented.

### The Ten-Group Classification System for Caesarean deliveries

The Ten-Group Classification System for CS deliveries provides a standardised framework for monitoring of obstetric practice for individual institutions. The classification is meant both for application to existing birth data, and for use as a prospective tool to identify at-risk groups. Contrary to previous classification systems for CS, the Ten-Group Classification System is independent of the medical indication(s) for a CS. Using this standardised classification it is easy to identify which groups are the primary contributors to the overall CS rate, as well as determine CS rates and pregnancy outcomes within the different obstetric groups. CS rates in each group and contributions to overall rate can be compared across different facilities and between different levels of facilities. It has been applied internationally in high-resource settings among equivalent sub-populations [14,19,20].

We applied the Ten-Group Classification System to existing birth data drawn from the medical birth registry at KCMC. We classified women into ten mutually exclusive groups based on four obstetric characteristics: previous obstetric history, gestational age, category of pregnancy and course of pregnancy [14].

### Demographic and medical variables

The essential information needed to apply the Ten-Group Classification System was available in the registry. We defined the following variables: parity coded as 0 or ≥ 1; multiple gestation coded as yes or no; presentation (at delivery) coded as cephalic, breech or abnormal; previous CS coded as yes or no; induction of labour coded as yes or no; CS coded as elective or non-elective; and gestational age coded as < 37 completed weeks or ≥ 37 completed weeks. We considered elective CS proxy for CS before labour, reflecting the practice at the facility. Gestational age was calculated according to the last menstrual period (LMP) registered on the antenatal card. For the 10% with missing LMP, birth weight ≥ 2,500 g was used as proxy for gestational age ≥ 37 weeks [21]. Information on the other variables necessary to complete the Ten-Group Classification System was missing in less than 1% of the sample.

Additional variables used to characterize the sample were: maternal age in years coded as < 20, 20-24, 25-29, 30-35 or > 35; parity coded as 0, 1-4 or ≥ 5; maternal education coded as none, primary (1-7 years), secondary (8-11 years) or higher (≥ 12 years); and current residence coded as rural, urban or semi-urban. Missing data were less than 1%. We selected medical characteristics known to be associated with adverse pregnancy outcome: female genital mutilation (FMG) coded as any type or none; HIV testing coded as recorded or not recorded, HIV status of those recorded coded as positive or negative; antenatal visits coded as 1-3 or ≥ 4; serious maternal morbidity (preeclampsia, eclampsia, abruptio placentae and placenta praevia) coded as yes or no; and low birth weight of < 2,500 g coded as yes or no.

We categorized the main admission diagnosis recorded among women delivered by CS. For formally-referred birthing women, this was the referral diagnosis, whilst for self-referred women the receiving midwife noted the main reason for arrival.

### Outcome variables

Selected maternal outcomes were maternal death, prolonged maternal hospital stay as proxy for maternal complications (> 9 days after day of delivery = 97.5 percentile) and major obstetric haemorrhage at delivery (≥ 750 mL). Data on haemorrhage by clinical estimation were available from 2005 onwards. Due to the high prevalence of anaemia among pregnant women in the area, we chose a cut-off of 750 mL as a clinically relevant level of obstetric haemorrhage [22]. Neonatal outcomes were neonatal death (excluding intrauterine death diagnosed before labour), low Apgar score (< 7 at 5 minutes) and postnatal transfer to the neonatal ward. We excluded cases with missing variables such as delivery mode or presentation (2%). The final sample included 20,662 births and 21,255 infants with complete information to enable classification using the Ten-Group Classification System.

### Referral classification

Women were categorized as formally-referred when they were referred by qualified health personnel from other hospitals or health facilities such as health centres or dispensaries. The criteria for referral of women for hospital delivery from other health facilities in Tanzania can be found in Table 1. Women who came directly to KCMC, bypassing referring facilities, were categorized as self-referred birthing women. The hospital charges these women an extra registration fee. Women delivered by CS in a previous pregnancy are routinely asked to register at KCMC for the next birth. These women were categorized as self-referred if not referred for other (medical or obstetric) reasons. Self-referred birthing women thus constituted a case mix of women with a wish to deliver in the facility (and able to pay), women directly seeking emergency assistance for obstetric complications bypassing referral facilities for whatever reason and women recommended for delivery at KCMC due to uterine scar(s) but without other obstetric complications. The hospital provides emergency transport for referrals between the regional birth centre (Mawenzi) and KCMC. From other facilities, transport was not regularly available. There were no community-based referral systems in place during the period. Missing referral status applied to 9.4% of the women. Demographic and obstetric characteristics and pregnancy outcomes for the missing cases were near identical to the total sample average (data not shown). These cases (n = 1950) were excluded from the outcome analysis.

**Table 1:** Criteria for referral from health facility to hospital-level delivery, Tz†

**Elective referral**	**Emergency intrapartum referral**
	
More than 4 pregnancies	Spontaneous rupture of membranes without labour
Height < 150 cm	Labour < 34 weeks
Pelvic deformity	Labour > 12 hours
First pregnancy at 35 or more years	Abnormal lie or presentation of baby
Previous Caesarean section or vacuum delivery	Vaginal bleeding
Previous postpartum haemorrhage	Abnormal foetal heart (< 120 or > 160)
Previous retained placenta	Elevated body temperature (> 38 degrees Centigrade)
Blood pressure > = 140/90 mm Hg	Eclampsia or blood pressure > = 140/90 mm Hg
Hb < 8.5 gm/dL	Hb < 8.5 gm/dL
Albuminuria	Small pelvis or big baby
Glucosuria	Meconium-stained amnion fluid
Gestational age > 40 weeks	
Intrauterine foetal death	
Abnormal lie (> 36 weeks)	
Oedema of legs, face and hands	
Suspected multiple gestation	
Fundal height not corresponding to gestational age	
Presence of danger signs*	

† According to the Reproductive and Child Health Card (RCHC-4), Ministry of Health, Tanzania. *As defined in the RCHC-4

### Details of ethics approval

Permission to conduct the study was granted by the National Institute for Medical Research of the Ministry of Health in Tanzania, and the ethics committee at KCMC Hospital. Approval date 2003, reg. NIMR/HQ/R:Sa/Vol. IX/126.

### Statistical analysis

We extracted and analyzed data with *Statistical Package for the Social Sciences/Predictive Analytics Software (SPSS/PASW) *version 16.0. We used the χ^2 ^test to determine trends in the proportion of CS and formally-referred birthing women during the period, and also to determine crude associations between referral status in CS deliveries and maternal/neonatal outcomes such as maternal/neonatal death, prolonged hospitalisation, obstetric haemorrhage, low Apgar score and transfer to the neonatal ward. Crude odds ratios (cOR) with corresponding 95% confidence intervals were estimated. We used a one-step multiple binary logistic regression framework to adjust the odds ratios and corresponding 95% confidence intervals for significant potential confounders such as type of CS, urban/rural residence, parity and low birth weight as proxy for preterm delivery. We considered significance level (p-value) below 0.05 statistically significant.

## Results

### Characteristics of the study population

Table 2 presents demographic, medical and outcome characteristics for the final sample of 20,662 women and 21,255 newborns. Multiple gestations comprised 2.9% of all deliveries. Of all women, 19% (N = 4,004) were formally referred. Among formally-referred birthing women, the majority (52%) came from the regional birth centre (Mawenzi) 3 km away. Demographic characteristics showed that the proportion of teenage mothers (13-19 years) was higher among formally-referred than self-referred birthing women (15.0% versus 8.5%). Formally-referred women were more frequently rural residents and had lower educational attainments than self-referred women (Table 2). A history of FGM, a diagnosis of serious maternal morbidity and unknown HIV status were also more prevalent among formally-referred women. As expected, formally-referred women had a higher rate of adverse outcomes such as low birth weight babies, maternal death, neonatal death, low Apgar score and transfer to the neonatal ward (Table 2). For other variables, there were no apparent major differences in characteristics according to referral status.

**Table 2:** Characteristics of 20,662 women/21,255 infants at KCMC, Tz, 2000-07

	All births		Referred		Self-referred	
			
	N = 20,662/21,255		N = 4,004/4,150		N = 14,708/15,041	
Factors	%	CS rate	%	CS rate	%	CS rate
†**Age, years (median 27)**						
< 20	9.6	29.7	15.0	45.7	8.5	22.2
20-24	27.2	30.2	27.9	53.2	27.6	23.9
25-29	28.7	33.0	23.8	59.3	30.0	27.4
30-35	24.3	35.3	22.0	58.7	24.5	29.9
> 35	10.1	35.6	11.4	55.5	9.4	30.6
†**Parity (mean 1.3, median 1)**						
0	38.5	28.9	40.3	48.8	39.0	23.1
1-4	58.1	35.6	55.0	60.4	58.2	29.7
> = 5	3.4	28.7	4.7	46.0	2.8	21.8
**Maternal education**						
None	2.2	45.2	4.2	58.7	1.7	35.7
Primary (1-7)	64.8	34.7	77.9	55.2	61.5	28.0
Secondary (8-11)	4.7	29.0	3.9	53.8	4.9	24.9
Higher (12+)	28.0	27.8	14.0	53.7	31.9	24.6
†**Current residence**						
Rural	46.2	38.1	67.2	53.4	40.2	31.9
Urban	49.3	27.4	27.9	57.9	55.3	23.1
Semi urban	4.5	36.3	4.9	60.7	4.5	29.6
**Female Genital Mutilation**						
FGM, any type	25.2	36.5	32.4	55.9	23.0	29.5
No FGM	74.8	31.5	67.6	54.7	77.0	26.2
**HIV status**						
†HIV test recorded	47.9	31.8	39.6	50.4	52.7	28.1
HIV + (of recorded)	6.4	32.4	7.3	44.8	6.3	29.5
**Antenatal care**						
One visit or more	99.2	32.7	99.2	55.1	99.3	26.9
4 visits or more	75.5	32.1	60.2	55.5	76.5	26.7
†Serious mat. morbidity*						
Yes	5.4	47.9	8.6	56.5	4.4	43.2
No	94.6	31.9	91.4	54.9	95.6	26.2
†**Low birth weight (g)**						
< 2500	13.1	32.2	18.5	49.4	11.6	33.6
> = 2500	86.9	38.6	81.5	56.1	88.4	26.4
**Outcomes**						
						
†Haemorrhage**						
> = 750 cc	2.3	81.3	3.5	83.3	2.0	80.4
< 750 cc	97.7	31.3	96.5	47.9	98.0	26.6
†**Postpartum stay**						
> 9 days	2.1	57.0	3.6	69.9	1.8	50.2
< = 9 days	97.9	31.7	96.4	54.3	98.2	26.0
†**Maternal death**						
Yes	0.1	40.7	0.2	37.5	0.1	44.4
No	99.9	32.7	99.8	54.9	99.9	26.9
†**Apgar score**						
Apgar < 7 at 5"	2.7	48.8	5.1	57.8	2.1	42.9
Apgar > = 7 at 5"	97.3	32.7	94.9	55.3	97.7	27.0
†**Neonatal death**						
Yes	1.0	44.0	1.9	46.8	0.7	43.1
No	99.0	32.8	98.1	54.9	99.3	27.0
†**Transfer to neonatal ward**						
Yes	14.2	21.8	21.5	61.9	12.2	45.3
No	85.8	10.5	78.5	52.7	87.8	24.6

†Associated to referral status (P < .01; Chi-Square). *Including hypertensive disorders in pregnancy including preeclampsia, abruptio placentae and placenta praevia. **Data available for 2005 and onwards.

Twenty-seven maternal deaths were recorded in the final sample, equivalent to a maternal mortality ratio (MMR) of 131/100,000 births. This does not reflect the true facility-based MMR, as deaths in early pregnancy, in other departments or postpartum were not routinely recorded in the registry. For all births the perinatal mortality rate was 44/1,000, of which stillborn rate was 38/1000 (44% fresh) and early (facility-based) neonatal death rate was 6/1,000, thus less than 1%. Only deaths occurring in the facility were included in the registry.

### Caesarean section rates in referred and self-referred birthing women

**Figure 1:**
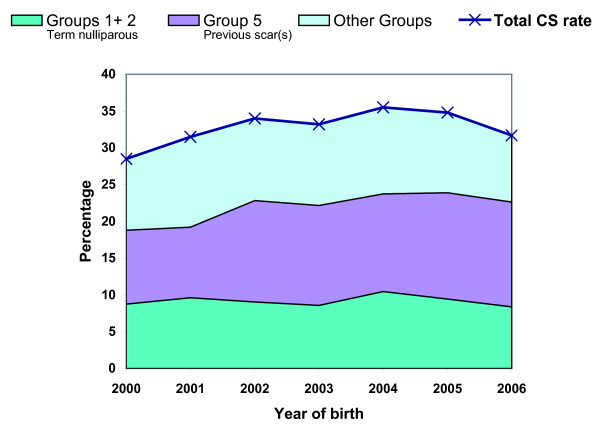
**Contribution of selected groups to overall CS rate, KCMC, 2000-06**.

In the final sample, 6,765 women were delivered by CS; a facility-based CS rate of 32.7%. Emergency CS constituted 80% of all CS deliveries. Less than 2% of all births were operative vaginal births (ventouse or forceps). CS rates rose from 28.5% in year 2000 to 35.5% in 2004, thereafter falling slightly to 31.7% in 2006 (Figure 1). Overall, there was a significant increase in CS rates over the study period (χ^2 ^for trend, *p *< 0.01). The proportion of formally-referred birthing women remained unchanged over the period (χ^2 ^for trend, *p *= 0.26).

Table 3 presents CS rates in the ten groups, sizes of the groups and their relative contributions to the overall rate for all births and by referral status. The proportion of CS was higher overall in the formally-referred group than in the self-referred group, 55% versus 27% (cOR 3.32, 95% CI 3.09-3.57, *p *< 0.001). Emergency CS was more frequent in the formally-referred than in the self-referred group, 85% versus 79% (cOR 1.50, 95% CI 1.30-1.72, *p *< 0.01). Elective CS rates were low in general, except in group 5 (previous scar) with singleton cephalic presentation) where both formally-referred and self-referred birthing women had rates > 20% (data not shown). For both referral categories women with previous scar(s) (group 5) and term singleton cephalic nulliparous women (groups 1 + 2) contributed most towards the total CS rate. These three groups comprised 66% of all CS in the facility (Figure 1).

**Table 3:** Caesarean section rates 2000-07 according to the Ten-Group Classification and referral status, KCMC, Tz

		Total CS rate (%)			Contrib. rate (%)		Size of group (%)	
				
Ten-Groups	N	Referred	**Self-refer**.	P*	Referred	**Self-refer**.	Referred	**Self-refer**.
1. Nulliparous, single ceph., > = 37 w, sp. labour	4,001	58.4	20.3	< .01	11.7	4.0	20.0	19.7
2. Nulliparous, single ceph., > = 37 w, induced/CS	2,877	32.9	24.5	< .01	4.2	3.6	12.8	14.6
3. Multiparous, single, ceph., > = 37 w, sp. labour	5,006	41.4	8.7	< .01	6.3	2.3	15.3	26.0
4. Multiparous, single, ceph., > = 37 w, induced/CS	2,079	32.5	15.7	< .01	2.2	1.7	6.8	10.7
5. Previous CS, single ceph., > = 37 w	3,556	82.6	69.7	< .01	19.3	10.8	23.4	15.5
6. Nulliparous breeches	135	74.4	65.9	.33	0.8	0.4	1.1	0.6
7. Multiparous breeches (incl. previous CS)	205	57.7	35.2	< .01	0.8	0.3	1.3	0.8
8. Multiple pregnancies (incl. previous CS)	589	46.4	36.8	.04	1.9	0.9	4.2	2.5
9. Abnormal lies (incl. previous CS)	60	93.1	85.7	.64	0.7	0.1	0.7	0.1
10. Single, ceph., < = 36 w (incl. previous CS)	2,154	49.5	30.3	< .01	7.1	2.8	14.3	9.4

**Total**	20,662	55.0	26.9	< .001	55.0	26.9	99.9	99.9

*Chi-squared/Fisher's Exact tests.

### Main admission diagnoses by referral status

Table 4 presents the main admission diagnoses by referral status for the 6,161 CS deliveries, as recorded in the registry. "Previous scar(s)" and "obstructed labour" were the most frequent registered diagnoses in both groups. In formally-referred birthing women these were followed by "cephalo-pelvic disproportion" and "poor progress," whilst in self-referred birthing women, these were followed by "poor progress" and "foetal distress."

**Table 4:** Main admission diagnosis recorded among 6,161 Caesarean births, KCMC, Tz

Diagnosis	Referred (N = 2,203)		Self-referred (N = 3,958)	
		
	N	%	N	%
1 previous scar +/- other obstetric diagnosis	430	19.5	469	11.8
2 previous scars +/- other obstetric diagnosis	222	10.1	186	4.7
3+ previous scars +/- other obstetric diagnosis	47	2.1	29	0.7
Abruptio	33	1.5	23	0.6
Anaemia/haematologic disorder	24	1.1	2	0.1
Ante partum haemorrhage	47	2.1	18	0.5
Bad obstetric history	29	1.3	23	0.6
Breech	52	2.4	70	1.8
Cephalo-pelvic disproportion	202	9.2	78	2.0
Eclampsia	53	2.4	27	0.7
Failure of trial of scar/induction	14	0.6	57	1.4
Foetal distress	123	5.6	155	3.9
Hypertension	31	1.4	20	0.5
Malpresentation/prolapse arm/cord	119	5.4	60	1.5
Multiple pregnancy	29	1.3	24	0.6
Obstructed labour	236	10.7	175	4.4
Placenta previa	36	1.6	23	0.6
Poor progress/prolonged labour	192	8.7	165	4.2
Post term	7	0.3	17	0.4
Preeclampsia	74	3.4	28	0.7
Preterm rupture membranes/preterm labour	30	1.4	42	1.1
Uterine rupture	8	0.4	6	0.2
Expected vaginal delivery	6	0.3	282	7.1
No diagnosis	121	5.5	1953	49.3
Other †	38	1.7	26	0.7

**Total**	**2,203**	**100**	**3,958**	**100**

†'Other' included maternal risk factor, diabetes, heart disease, cardiac arrest, HAART, PPH, chorio chorioamnionitis, IUFD, vaginal warts, malformation baby, repaired fistula, diarrhoea, pelvic mass, IUGR, cervix cancer, difficult labour, mental disease, physical handicap, fracture.

### Associations between referral status and post-Caesarean section maternal and neonatal outcomes

Table 5 presents the effect of referral status on cOR and adjusted odds ratio (aOR) for six maternal and neonatal outcomes after CS. By univariate analyses, neither maternal death (N = 7, cOR 1.35, 95% CI 0.30-6.06, *p *= 0.71) nor obstetric haemorrhage (cOR 1.05, 95% CI 0.73-1.52, *p *= 0.78) was associated with referral status. Prolonged postpartum stay was associated with formally-referred status (cOR 1.38, 95% CI 1.05-1.81, *p *= 0.02), but this effect did not remain significant after adjusting for potential confounders such as parity, type of CS, preterm birth and residence.

**Table 5:** Associations between referral status and pregnancy outcomes in Caesarean births, KCMC, Tz

	All CS		Referred		**Self-ref**.		cOR	CI for cOR	*P*	aOR†	CI for aOR†	a*P*†
									
Maternal outcomes	N	%	N	%	N	%						
Maternal death	7	0.1	3	0.1	4	0.1	1.35	0.30-6.06	.71	NI		
Haemorrhage*	135	5.8	45	6.0	90	5.7	1.05	0.73-1.52	.78	NI		
Prolonged stay	220	3.8	95	4.5	125	3.3	1.38	1.05-1.81	.02	1.22	0.92-1.61	.17
**Neonatal outcomes**												

Neonatal death	83	1.3	36	1.6	47	1.2	1.39	0.90-2.16	.14	1.37	0.87-2.16	.17
Low Apgar	250	4.0	118	5.3	132	3.3	1.65	1.28-2.13	.00	1.42	1.09-1.86	.01
Transfer neonatal ward	1388	21.9	553	24.4	835	20.5	1.25	1.11-1.41	.00	1.18	1.04-1.35	.01

†Adjusted for parity, low birth weight, type of CS, residence. *Data from 2005 onwards. NI = Not included in multiple regression analysis.

Analysis of neonatal outcomes after CS did not find any association between formal referral and neonatal death (aOR 1.37, 95% CI 0.87-2.16). Both low Apgar score (aOR 1.42, 95% CI 1.09-1.86, *p *< 0.01) and transfer to the neonatal ward (aOR 1.18, 95% CI 1.04-1.35, *p *< 0.01) were associated with formal referral after adjusting for parity, low birth weight, type of CS and residence. Maternal age was not associated with the outcomes by univariate analysis and was not included as an adjusting factor.

### Outcomes after Caesarean section by referral status in the Ten-Group Classification System

Tables A1 and A2 (Additional file 1) show the distribution of adverse maternal and perinatal outcomes in CS deliveries according to The Ten-Group Classification System. The absolute number of cases per group is small, and data must be interpreted with caution. Obstetric haemorrhage occurred in one out of ten preterm CS deliveries (group 10), and was also prevalent in other obstetric high-risk groups (groups 6-9). Neonatal death rates were > 2% in group 3 (multiparous, spontaneous labour), groups 6 and 7 (breech), group 8 (multiple gestation) and group 10 (preterm singleton cephalic). Frequency of low Apgar score was > 7% in groups 6 and 7 (breech), group 9 (malpresentation) and group 10 (preterm). Among formally-referred CS, low Apgar score was prevalent in groups 6 and 7 (breech) and group 10 (preterm). Among self-referred CS, low Apgar score was most prevalent in group 10 (preterm). Transfer to the neonatal ward occurred in one out of four formally-referred births, compared with one out of five self-referred births. Both formally-referred and self-referred births in group 5 (previous scar) had low frequencies of obstetric haemorrhage, neonatal death and low Apgar score.

### Analysis of results before and after introduction of cost-sharing

Due to the gradual introduction of cost-sharing from 2005 (during the period under study), we compared results from before and after January 1st 2005.

There was no difference in the proportion of formally-referred birthing women between the time periods (21.5% versus 21.1%, p < 0.57). Formally-referred women were significantly younger, with a lower level of education and a higher proportion of rural residents in the second time period compared with the first. Self-referred women on the contrary were significantly older, had better educational attainments and a lower proportion of rural residents in the second time period. In both groups the proportion with four or more antenatal visits decreased significantly between the first time period and the second.

The overall CS rate among formally-referred birthing women fell from 58.8% to 49.2% between the first time period and the second (OR 0.68, CI 0.60-0.78, p < 0.001), whilst the rate among self-referred women remained unchanged. The main reasons for referral were the same for the two time periods. For CS births, only neonatal death showed a crude association with the time period, with an increase of overall neonatal deaths from 0.9% to 1.2% (p < 0.03) between the first time period and the second. Adding time period as an explanatory variable in the regression models did not impact on the adjusted estimates.

## Discussion

Based on registry data from 21,000 hospital-based births at a tertiary care centre in northeastern Tanzania, we found that 80% of birthing women were self-referred and only 20% were formally referred through the national referral system. Formal referral was associated with rural residence, less education and a higher obstetric risk profile. As expected, CS rates were higher in formally-referred than self-referred women, but the main contributing groups (women with previous CS and nulliparous women) were the same regardless of referral status. Overall there was a high repeat CS rate and a low prevalence of operative vaginal delivery, both of which affect utilization of CS. Low Apgar score and transfer to the neonatal ward were both associated with formal referral for delivery. Poor neonatal outcome rates were high in some subgroups (breech, multiple gestation and preterm birth) of self-referred CS deliveries, pointing to a significant proportion of risk in women that bypass or are not reached by referral systems. Conversely, we found no differences in adverse maternal outcomes after CS in formally-referred versus self-referred birthing women.

In this study, we have shown that basic data, which are already collected in most delivery wards, can be usefully evaluated in a standardised way. The strength of this study lies in the large sample size and completeness of data for the majority of CS deliveries conducted in the area. A small number of CS deliveries performed elsewhere in the area were not included, but the magnitude of their effects on our results would be small. Using registry data imposed limitations on our study. Although validity checks are performed regularly, registry data are likely to be of lesser quality than data specifically collected for research purposes. We did not have direct information from the referring facilities nor referring health staff to be able to assess the compliance, appropriateness and timeliness of referral. We also did not have data regarding community referral processes such as decision-making in the event of an obstetric complication, or information about women that never reached appropriate care. Such data would have added value to the study. Multiple levels of referral and delay in receiving care once in the tertiary care centre could not be assessed, as this information was not included in the registry. Proxy endpoints such as infection parameters or blood transfusion were not part of the registry information. This limited our selection of outcomes. Patient selection played a role as only a proportion of women in Kilimanjaro will reach a tertiary facility in the event of complications. In particular, out-of-pocket fees are likely to be a barrier to seeking care, which is supported by our finding that women giving birth at KCMC have better educational attainments compared with the regional average [6]. Measures of association between referral status and outcome are less likely to be influenced by selection bias.

Interestingly, the groups that contributed the most to overall CS rates were the same in formally-referred and self-referred women. Comparing with Ten-Group Classification System data from the US and Europe, we found similar CS rates in groups 1 + 2 (term singleton cephalic nulliparous women), and in contributions of groups 1 + 2 + 5 to the total rate [23]. Similarly, we found that the high CS rate in term singleton cephalic nulliparous women contributes to a high overall CS rate, similar to high-resource contexts [23]. We also found dissimilarities, such as high CS rates among self-referred women in groups 1 and 3 (spontaneous labour) compared with groups 2 and 4 (induced or elective CS before labour) in our setting compared with high-resource contexts. This suggests a failure to detect obstetric morbidity at antenatal care for induction or planned CS, allowing high-risk cases to proceed into labour. Group 5 (previous scar) was responsible for 40% of CS cases in self-referred women. We believe the high repeat CS rate in this group of 70% was affected by a shortage of attempts at trial of labour due to lack of human and material resources. By definition, this subgroup had no other registered obstetric complication, explaining the favourable fetal outcomes following CS. Use of ventouse or forceps was not part of the resident training programme in this facility, which will increase CS rates in most obstetric groups, compared with other settings.

Although not directly comparable, the hospital-based early perinatal mortality rate was high (4.4%) compared with high-resource settings where population-based perinatal mortality rate is often as low as 0.5% [24]. The early (hospital-based) neonatal mortality rate in babies born by CS of 1.3% in this facility reflects not only obstetric conditions, but also lack of advanced intensive care neonatal services. Investigation of differences between subgroups within the self-referred population showed that group 5 (previous scar) had low rates of all adverse neonatal outcomes, indicating that these risks lie within other obstetric groups. The surprising finding that adverse maternal outcomes were not related to referral status is likely due to the proximity of the main referring facility with provision of emergency transport. Thus mothers that make it to the referral centre receive CS in time to save their lives. Although we do show that rural and otherwise disadvantaged women have some degree of access to referral, the low absolute number of referrals from rural facilities confirms a disappointing underuse of CS for rural Tanzanian women [13]. Among self-referred women, women with no education and with rural residence had the highest CS rates, strongly suggesting the need to target this subgroup for earlier professional care and referral. Jahn et al. identified substandard antenatal care and lack of community trust in lower level health providers as contributing factors to self-referral in southern Tanzania [25]. After the introduction of user fees, we found a less favourable sociodemographic profile in formally-referred women, whilst the opposite was the case for self-referred women. A selection bias towards referral of poor women that are eligible for exemption from fees could explain these findings. We found that the proportion of women accessing the recommended four antenatal visits decreased between the first time period and the second. Reversing this trend is necessary to improve third trimester detection of increased risk conditions with poor outcomes such as breech, multiple gestation and threatened preterm labour, thus assuring earlier referral for optimal care. We still need more operational knowledge of utilization of services by women and babies at risk to expand equitable access.

To better evaluate the effects of reproductive health programs, we would advocate for the expansion of facility registers to regional-based birth registries. This would give better return on investment than single registers in a situation where a countrywide population-based health information system is not yet feasible. The Ten-Group Classification System is widely used in high-resource settings, but so far less used in low-resource settings. This could be due to limited resources available for systematic CS audits. We believe it offers a standardized tool that, combined with knowledge of local obstetric epidemiology, is useful in the continuous evaluation of regional obstetric care and hence promotion of quality and equity.

## Conclusions

Women formally referred for delivery constituted a low proportion of all births, but contributed substantially to CS rates and adverse outcomes, indicating that the referral system does identify high-risk women. Nevertheless, subgroups within the larger population of self-referred women showed poor neonatal outcomes in absolute terms, suggesting a dire lack of timely identification. Strategies to improve neonatal outcomes after CS deliveries must strengthen both the existing referral system and cater to subgroups of self-referred births not caught by the present system. Targeted interventions should be directed towards improving the continuum of care for obstetric risk groups, especially multiple gestation, breech and preterm births. To be able to promote rational delivery of care and evaluate the effectiveness of operational interventions in this context, regional information gathering systems would provide a much better return on investment than single facility-based registers.

## Competing interests

The authors declare that they have no competing interests.

## Authors' contributions

IKS, SV and PB conceived of the study and participated in its design. IS conducted the statistical analyses and drafted the manuscript in collaboration with SV. OO participated in design of the study, interpretation of data and coordination of the work. All authors read and commented on drafts of the manuscript. All authors except PB approved the final manuscript.

## Pre-publication history

The pre-publication history for this paper can be accessed here:

http://www.biomedcentral.com/1471-2393/11/55/prepub

## Supplementary Material

Additional file 1 **Tables A1 and A2**. Table A1. Ten-Group Classification versus maternal outcomes in 6,161 Caesarean sections. with known referral status, KCMC, Tz. Table A2. Ten-Group Classification versus neonatal outcomes in 6,388 Caesarean births, with known referral status, KCMC, Tz. Click here for file
