# Supplementary material for: Caesarean section among referred and self-referred birthing women: a cohort study from a tertiary hospital, northeastern Tanzania
Source: BMC Pregnancy Childbirth. 2011 Jul 28;11:55. doi: 10.1186/1471-2393-11-55 (PMC3160415; doi:10.1186/1471-2393-11-55)
Supplement: Additional file 1 — Tables A1 and A2. Table A1. Ten-Group Classification versus maternal outcomes in 6,161 Caesarean sections. with known referral status, KCMC, Tz. Table A2. Ten-Group Classification versus neonatal outcomes in 6,388 Caesarean births, with known referral status, KCMC, Tz. [file 1471-2393-11-55-S1.DOC]

| **Table A1** | | | | | | | | | | | | | |  |
| --- | --- | --- | --- | --- | --- | --- | --- | --- | --- | --- | --- | --- | --- | --- |
|  | | | | | | | | | |  |  |  |  |  |
|  | **Maternal death** | | | |  | **Haemorrhage** | | | |  | **Prolonged postp. stay** | | | |
|  | **Referred** | | **Self-ref.** | |  | **Referred** | | **Self-ref.** | |  | **Referred** | | **Self-ref.** | |
| **Ten-Groups** | N | % | N | % |  | N | % | N | % |  | N | % | N | % |
| **1** | 1 | 0.2 | 3 | 0.5 |  | 4 | 2.3 | 6 | 2.9 |  | 18 | 4.1 | 22 | 4.0 |
| **2** | 0 | 0 | 0 | 0 |  | 2 | 3.2 | 10 | 5.5 |  | 5 | 3.1 | 13 | 2.6 |
| **3** | 1 | 0.3 | 0 | 0 |  | 6 | 6.3 | 8 | 6.4 |  | 13 | 5.3 | 9 | 2.8 |
| **4** | 0 | 0 | 0 | 0 |  | 1 | 3.3 | 6 | 6.9 |  | 0 | 0 | 8 | 3.4 |
| **5** | 0 | 0 | 0 | 0 |  | 14 | 5.8 | 33 | 4.5 |  | 24 | 3.3 | 26 | 1.7 |
| **6** | 0 | 0 | 0 | 0 |  | 0 | 0 | 1 | 7.1 |  | 3 | 10.0 | 0 | 0 |
| **7** | 0 | 0 | 0 | 0 |  | 2 | 22.2 | 0 | 0 |  | 1 | 3.6 | 1 | 2.5 |
| **8** | 0 | 0 | 0 | 0 |  | 2 | 8.0 | 7 | 17.9 |  | 7 | 9.2 | 9 | 7.1 |
| **9** | 0 | 0 | 0 | 0 |  | 1 | 33.3 | 0 | 0 |  | 1 | 3.7 | 1 | 5.9 |
| **10** | 1 | 0.4 | 1 | 0.2 |  | 13 | 12.1 | 19 | 10.7 |  | 23 | 8.5 | 36 | 9.1 |
| **Total** | 3 | 0.1 | 4 | 0.1 |  | 45 | 6.0 | 90 | 5.7 |  | 95 | 4.5 | 125 | 3.3 |
|  |  |  |  |  |  |  |  |  |  |  |  |  |  |  |
|  |  |  |  |  |  |  |  |  |  |  |  |  |  |  |
| **Table A2** | | | | | | | | | | | | | |  |
|  | | | | | | | | | |  |  |  |  |  |
|  | **Neonatal death** | | | |  | **Low Apgar at 5"** | | | |  | **Transfer NICU** | | | |
|  | **Referred** | | **Self-ref.** | |  | **Referred** | | **Self-ref.** | |  | **Referred** | | **Self-ref.** | |
| **Ten-Groups** | N | % | N | % |  | N | % | N | % |  | N | % | N | % |
| **1** | 8 | 1.7 | 7 | 1.2 |  | 34 | 7.3 | 30 | 5.1 |  | 148 | 31.8 | 124 | 21.3 |
| **2** | 2 | 1.2 | 5 | 1.0 |  | 12 | 7.1 | 9 | 1.7 |  | 42 | 25.1 | 108 | 20.6 |
| **3** | 5 | 2.0 | 9 | 2.7 |  | 16 | 6.4 | 16 | 4.9 |  | 68 | 27.1 | 83 | 25.2 |
| **4** | 2 | 2.2 | 3 | 1.2 |  | 1 | 1.1 | 6 | 2.5 |  | 22 | 24.7 | 43 | 17.6 |
| **5** | 3 | 0.4 | 5 | 0.3 |  | 19 | 2.5 | 16 | 1.0 |  | 95 | 12.4 | 199 | 12.5 |
| **6** | 0 | 0 | 2 | 3.8 |  | 3 | 9.4 | 3 | 5.8 |  | 12 | 38.7 | 11 | 21.2 |
| **7** | 1 | 3.6 | 1 | 2.3 |  | 2 | 8.0 | 3 | 7.3 |  | 9 | 32.1 | 11 | 25.0 |
| **8** | 4 | 2.5 | 6 | 2.2 |  | 7 | 4.4 | 10 | 3.7 |  | 43 | 26.7 | 76 | 27.6 |
| **9** | 0 | 0 | 0 | 0 |  | 2 | 9.1 | 1 | 5.6 |  | 10 | 37.0 | 8 | 44.4 |
| **10** | 11 | 4.0 | 9 | 2.2 |  | 22 | 8.3 | 38 | 9.5 |  | 104 | 37.0 | 172 | 41.2 |
| **Total** | 36 | 1.6 | 47 | 1.2 |  | 118 | 5.3 | 132 | 3.3 |  | 553 | 24.4 | 835 | 20.5 |
